# Supplementary material for: Association between oral microbiome and seven types of cancers in East Asian population: a two-sample Mendelian randomization analysis
Source: Front Mol Biosci. 2023 Nov 21;10:1327893. doi: 10.3389/fmolb.2023.1327893 (PMC10702768; doi:10.3389/fmolb.2023.1327893)
Supplement: Supplementary file 1 [file DataSheet1.ZIP › Supplementary Materials/MR plots for tongue/tongue═╝/Lung cancer/pheno.90_to_lung cancer_forest.pdf]

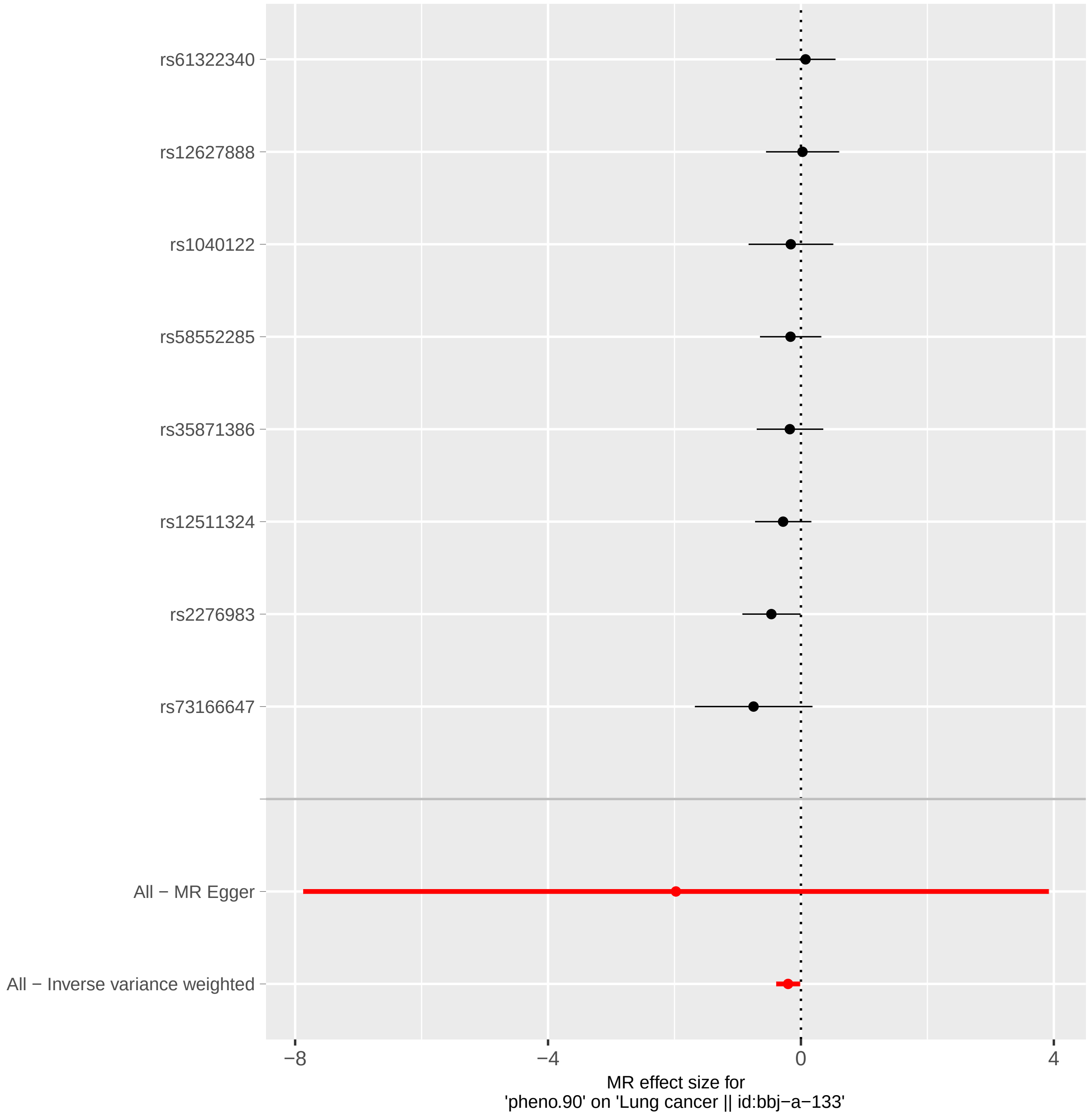
rs61322340
rs12627888
rs1040122
rs58552285
rs35871386
rs12511324
rs2276983
rs73166647
All − MR Egger
All − Inverse variance weighted
−8
−4
0
4
MR effect size for
'pheno.90' on 'Lung cancer || id:bbj−a−133'
